# Antiglioma Potential of Coumarins Combined with Sorafenib

**DOI:** 10.3390/molecules25215192

**Published:** 2020-11-08

**Authors:** Joanna Sumorek-Wiadro, Adrian Zając, Ewa Langner, Krystyna Skalicka-Woźniak, Aleksandra Maciejczyk, Wojciech Rzeski, Joanna Jakubowicz-Gil

**Affiliations:** 1 Department of Functional Anatomy and Cytobiology, Maria Curie-Sklodowska University, Akademicka 19, 20-033 Lublin, Poland; joanna.sumorek@gmail.com (J.S.-W.); a.zajac.umcs@gmail.com (A.Z.); olamaciejczyk77@gmail.com (A.M.); rzeskiw@hektor.umcs.lublin.pl (W.R.); 2 Department of Medical Biology, Institute of Rural Health, Institute of Agricultural Medicine, Jaczewskiego 2, 20-950 Lublin, Poland; ewa.langner@gmail.com; 3 Independent Laboratory of Natural Products, Medical University of Lublin, Chodzki 1, 20-093 Lublin, Poland; kskalicka@pharmacognosy.org

**Keywords:** osthole, umbelliferone, esculin, 4-hydroxycoumarin, sorafenib, apoptosis, autophagy

## Abstract

Coumarins, which occur naturally in the plant kingdom, are diverse class of secondary metabolites. With their antiproliferative, chemopreventive and antiangiogenetic properties, they can be used in the treatment of cancer. Their therapeutic potential depends on the type and location of the attachment of substituents to the ring. Therefore, the aim of our study was to investigate the effect of simple coumarins (osthole, umbelliferone, esculin, and 4-hydroxycoumarin) combined with sorafenib (specific inhibitor of Raf (Rapidly Accelerated Fibrosarcoma) kinase) in programmed death induction in human glioblastoma multiforme (T98G) and anaplastic astrocytoma (MOGGCCM) cells lines. Osthole and umbelliferone were isolated from fruits: *Mutellina purpurea* L. and *Heracleum leskowii* L., respectively, while esculin and 4-hydroxycoumarin were purchased from Sigma Aldrich (St. Louis, MO, USA). Apoptosis, autophagy and necrosis were identified microscopically after straining with specific fluorochromes. The level of caspase 3, Beclin 1, PI3K (Phosphoinositide 3-kinase), and Raf kinases were estimated by immunoblotting. Transfection with specific siRNA (small interfering RNA) was used to block Bcl-2 (B-cell lymphoma 2), Raf, and PI3K expression. Cell migration was tested with the wound healing assay. The present study has shown that all the coumarins eliminated the MOGGCCM and T98G tumor cells mainly via apoptosis and, to a lesser extent, via autophagy. Osthole, which has an isoprenyl moiety, was shown to be the most effective compound. Sorafenib did not change the proapoptotic activity of this coumarin; however, it reduced the level of autophagy. At the molecular level, the induction of apoptosis was associated with a decrease in the expression of PI3K and Raf kinases, whereas an increase in the level of Beclin 1 was observed in the case of autophagy. Inhibition of the expression of this protein by specific siRNA eliminated autophagy. Moreover, the blocking of the expression of Bcl-2 and PI3K significantly increased the level of apoptosis. Osthole and sorafenib successfully inhibited the migration of the MOGGCCM and T98G cells.

## 1. Introduction

Coumarins, classified as secondary metabolites, constitute a large group of ubiquitous compounds in the plant world. Depending on the chemical structure, simple coumarins, pyranocoumarins, and furanocoumarins can be distinguished [1,2].

Coumarin derivatives exhibit a wide spectrum of biological activity. Research conducted to date indicates a beneficial effect of coumarins on the central nervous system (analgesic, anticonvulsant, antidepressant, and sedative) and the circulatory system (anticoagulant and antihypertensive effect). They show antioxidant, antibacterial, antifungal, anti-inflammatory, antiallergic, and antiviral activity. Their antitumor activity is particularly important as well. These compounds have been shown to act at various stages of carcinogenesis. They exhibit chemopreventive properties as well as cytotoxic and antiproliferative activity against cancer cells. In addition, they limit angiogenesis and prevent the formation of metastases to other tissues [1,2,3,4,5]. Clinical studies have shown that coumarins have promising activity against several types of cancer, such as breast cancer, lung cancer, malignant melanoma, prostate cancer and renal cell carcinoma [6,7]. Simple coumarin derivatives improved the health condition of patients and did not show any toxic properties. Renal cell carcinoma patients tolerated a wide spectrum of coumarin doses, and the most common side effect was nausea associated with the intense aroma of the compound [7,8]. Interestingly, previous studies have also shown that coumarins may be used not only in the treatment of cancer but also in the treatment of the side effects of radiation therapy, such as radiogenic sialadenitis and mucositis [9].

**Figure 1:**
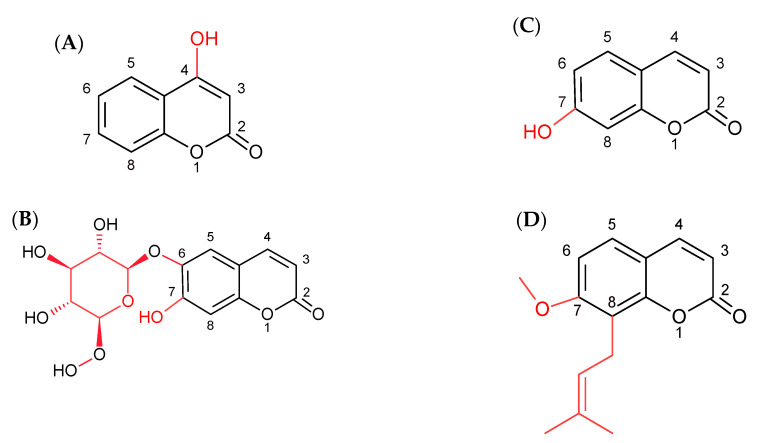
Structure of 4-hydroxycoumarin (**A**), esculin (**B**), umbelliferone (**C**) and osthole (**D**).

The cytotoxicity of coumarins towards cancer cells depends on their chemical structure; therefore, knowledge of the effect of various substituents on the antitumor properties of these compounds will ensure in more effective plans of therapeutic strategies. Special attention has been paid to simple coumarins, e.g., 4-hydroxycoumarin, umbelliferone, esculin, and osthole, differing in their location or the type of attached substituents (Figure 1). 4-hydroxycoumarin and umbelliferone (7-hydroxycoumarin) are isomers with a hydroxyl moiety located at the C4 and C7 positions of the coumarin ring, respectively. Esculin (6,7-dihydroxycoumarin 6-glucoside) is an analogue of umbelliferone with an additional glycosidic moiety at the C6 position. In turn, osthole (7-metoxy-8-isopentenyl-coumarin) has a methoxy moiety at the C7 position and an isoprenyl substituent at the C8 position [10].

It has been shown that all these hydroxycoumarins have the ability to reduce the proliferation, adhesion, and migration of cancer cells. Esculin interferes with the adhesion of U87 glioblastoma cells by modulating the function of integrins [11]. 4-hydroxycoumarin disorganizes the actin cytoskeleton in B16-F10 melanoma cells and reduces the potential of this tumor to metastasize to the lungs, as shown in mice [12,13,14]. Umbelliferone, in turn, reduces the migration of laryngeal cancer (RK33) and rat breast adenocarcinoma (RBA) cells [15,16]. 7-hydroxycoumarin has cytotoxic properties against many human cell lines such as leukemia (HL-60) and lung (A549 and H727), kidney (ACHN), and breast cancers (MCF-7) [17]. This compound inhibits the G1 phase cell cycle in human renal cell carcinoma cells (786-O, OS-RC-2, and ACHN) by reducing the expression of proteins that positively regulate the cell cycle (CDK2, CyclinE1, CDK4, and CyclinD1). Moreover, it modulates the expression of proteins involved in apoptosis (Bax and Bcl-2) and in proliferation (Ki67) [18].

Similar to hydroxycoumarins, osthole inhibits the migration (MCF-7) and invasiveness of breast cancer cells (MDA-MB-231BO) [19]. Additionally, it inhibits proliferation (in lung cancer: A549, leukemia: P-388 D1, breast carcinoma: MCF-7 and MDA-MB 231, medulloblastoma: TE671 and larynx carcinoma: RK33) by inducing apoptosis and inhibiting the cell cycle in the G2/M phase. At the molecular level, it is associated with a decrease in the expression of proteins involved in the cell cycle (CDK2, CyclinB1) and activation of apoptotic proteins (caspase 3, caspase 9, caspase 8, p53 protein) [20,21,22,23,24].

Anaplastic astrocytoma (AA, grade III) and glioblastoma multiforme (GBM, grade IV) are malignant tumors of the central nervous system. At the molecular level, they are characterized by the presence of mutations within genes, the products of which are involved in enhancement of intracellular signal transmission from the cell membrane to the nucleus. This applies in particular to the prosurvival pathways responsible for the regulation of cell proliferation and differentiation: Ras/MEK/ERK (Ras-Ras protein, MEK—mitogen-activated protein kinase, ERK—extracellular signal-regulated kinase) and PI3K/Akt/mTOR (PI3K-phosphoinositide 3-kinase, Akt/PKB-protein kinase B, mTOR- mammalian target of rapamycin kinase). It has been described that blocked signal transmission may be beneficial in enhancement of glioma cell sensitivity. It is also known that combination therapy, especially with the use of natural compounds, can increase the anticancer potential of clinically used pharmacotherapy [25,26].

Therefore, in our research, the antiglioma effect of simple coumarins (4-hydroxycoumarin, umbelliferone, esculin, and osthole) in combination therapy with sorafenib (Raf kinase inhibitor) was evaluated in terms of programmed cell death induction and migratory potential. At the molecular level, these processes were confirmed by the level of caspase 3, Beclin 1, Raf, and PI3K expression. Direct involvement of these proteins in apoptosis, autophagy, and mobility was studied by blocking their expression by specific siRNA.

## 2. Results

### 2.1. Effect of Simple Coumarins (Osthole, Esculin, Umbelliferone, or 4-Hydroxycoumarin) in Combination with Sorafenib on Apoptosis, Necrosis, and Autophagy Induction

**Figure 2:**
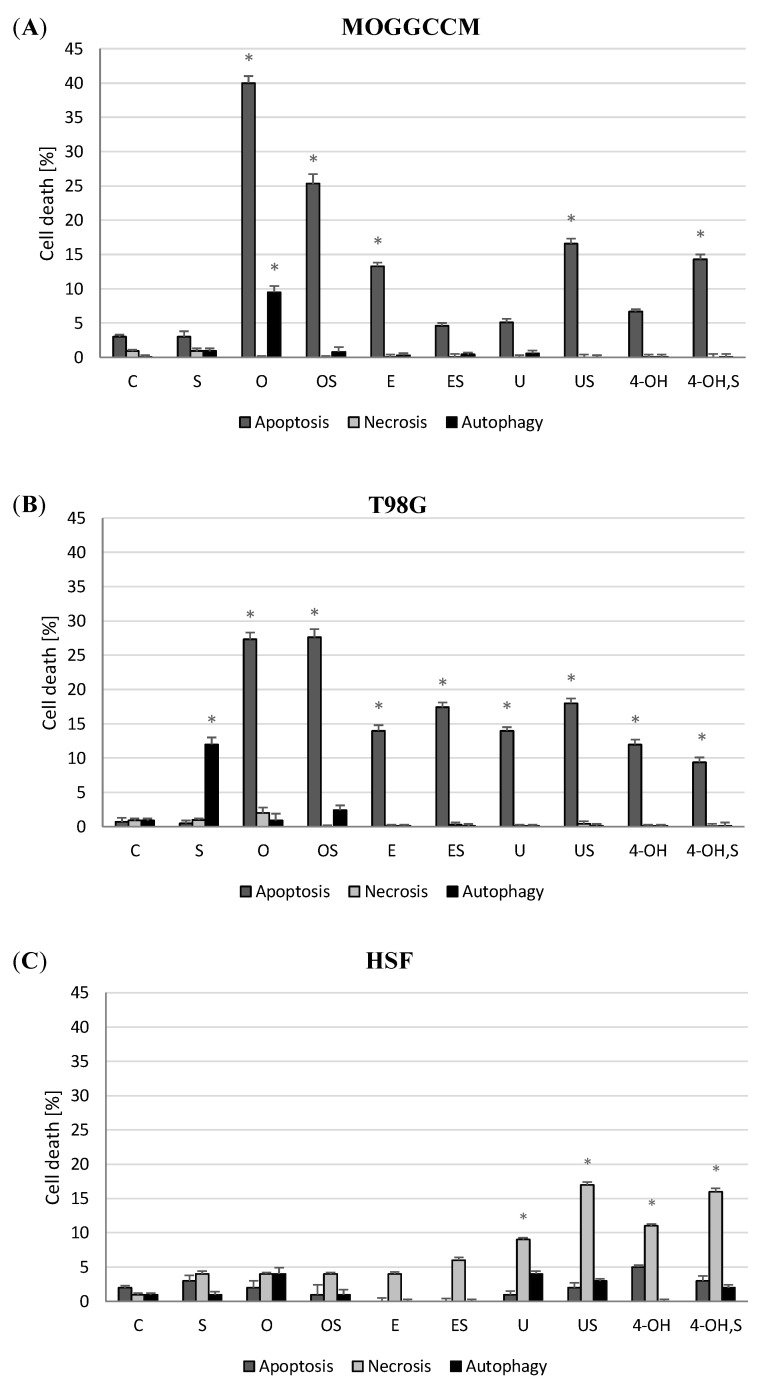
Effect of sorafenib and osthole, esculin, umbelliferone or 4-hydroxycoumarin administered separately or simultaneously on apoptosis, necrosis, and autophagy induction in the MOGGCCM (**A**), T98G (**B**) and HSF (Human Skin Fibroblasts) (**C**) cell lines. C—control, S—sorafenib, O—osthole, E—esculin, U—umbelliferone, 4-hydroxycoumarin; * *p* < 0.05.

Our research shows that all the coumarins effectively eliminated tumor cells by apoptosis. Osthole was the most effective compound in both cell lines, as it induced this type of death in 40% and 30% in AA and GBM, respectively (Figure 2A,B). Moreover, the coumarin-induced autophagy (10%) in the MOGGCCM cell line. The application of umbelliferone, esculin, and 4-hydroxycoumarin led to apoptosis in approximately 15% of cells in the T98G cell line (Figure 2B). Slightly different results were obtained in the case of the MOGGCCM cell line (Figure 2A). It turned out to be less sensitive to the action of umbelliferone and 4-hydroxycoumarin, which initiated apoptosis at a level lower than 7%.

Sorafenib had no significant effect on the induction of apoptosis but initiated autophagy in approx. 15% of the T98G cells (Figure 2B). The simultaneous application with the coumarins did not potentiate such an anticancer activity effectively. In the MOGGCCM line, sorafenib diminished the antiglioma potential of osthole, inducing apoptosis in ca. 25% and autophagy in 1% of cells in comparison to the single application of the coumarin. Similar effects were obtained upon the application of esculin, which in combination with sorafenib also showed lower proapoptotic activity. Interestingly, the simultaneous treatment with the hydroxycoumarins and sorafenib was more effective, causing apoptosis in up to 17% of cells. 

The experiments carried out on primary human skin fibroblasts (HSF) showed that osthole and esculin (alone and in combination with sorafenib) did not exert cytotoxic effects against normal cells. Different results were obtained for the hydroxycoumarins, as they showed a strong necrotic effect (nearly 20%), which was additionally enhanced by the addition of sorafenib. For this reason, osthole was chosen for further experiments.

### 2.2. Effect of Osthole and Sorafenib on the Migration Potential of Neoplastic Cells

**Figure 3:**
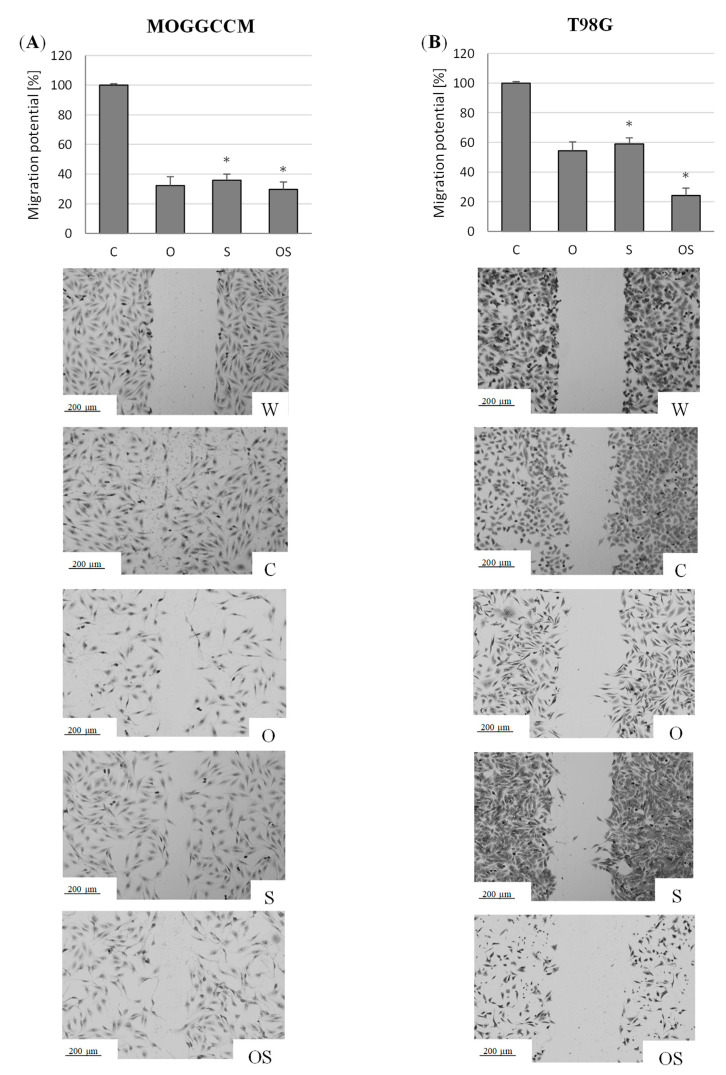
Migration potential of the MOGGCCM (**A**) and T98G (**B**) cells upon osthole and sorafenib treatment presented as the percent of cells within the wound. W—wound, C—control, O—osthole, S—sorafenib; * *p* < 0.05.

Inhibition of tumor cell migration plays an important role in anticancer therapy. The wound healing test showed that the treatment with osthole, sorafenib, and the combination of these compounds significantly decreased the migration potential of the AA and GBM cells (Figure 3). The combination therapy was the most effective, as it lowered this activity by approx. 70% compared to the control in both cell lines.

### 2.3. Effect of Osthole and Sorafenib on the Expression of Cell Death Marker Proteins

#### 2.3.1. Expression of Caspase-3, PI3K, and Raf Kinases

**Figure 4:**
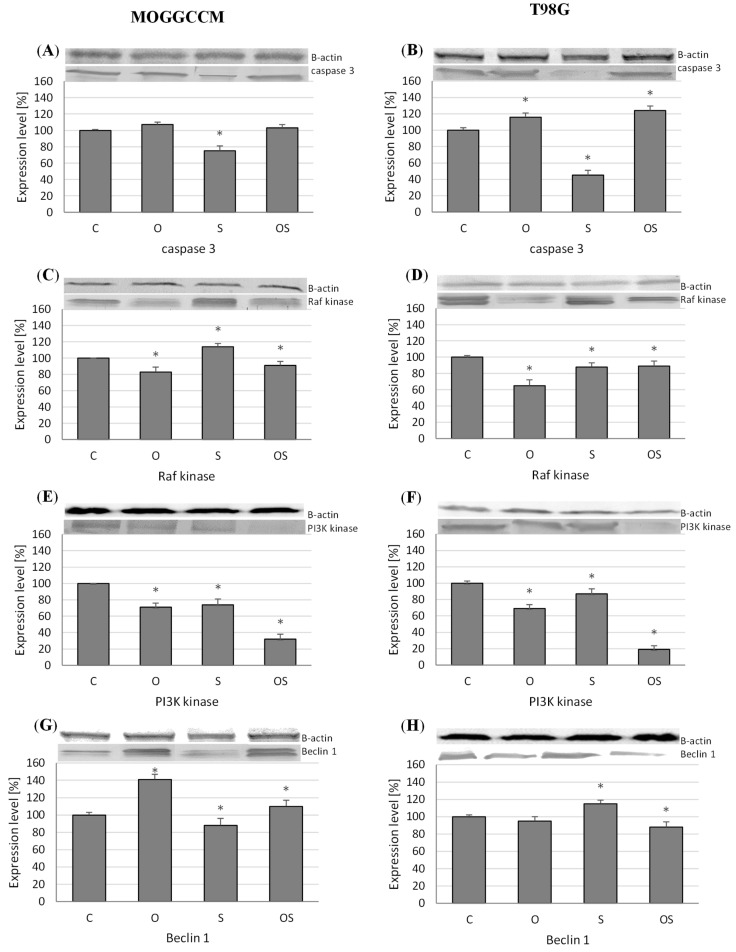
Effect of osthole, sorafenib and combined treatment with both drugs on the expression of caspase 3 (**A**,**B**), PI3K (Phosphoinositide 3-kinase) (**C**,**D**), Raf (Rapidly Accelerated Fibrosarcoma) (**E**,**F**), and Beclin 1 (**G**,**H**) in the MOGGCCM (**A**,**C**,**E**,**F**) and T98G (**B**,**D**,**G**,**H**) cell lines. C—control, O—osthole, S—sorafenib; * *p* < 0.05.

Caspase 3 is a member of the cysteine-aspartic acid protease family playing a key role in the execution phase of apoptosis. Our studies showed that the use of osthole alone and in combination with sorafenib led to an increase in caspase 3 expression in both cell lines (Figure 4A,B). The best effects (a 14% increase) were obtained upon the administration of osthole in combination with sorafenib in the T98G line. Moreover, the treatment with sorafenib alone reduced the level of this protein by 25% in the MOGGCCM line and by 55% in the T98G line.

PI3K and Raf kinases are also involved in the course of apoptosis and promote the survival of tumor cells. The Western blot analysis showed that osthole alone and in combination with sorafenib decreased the level of Raf kinase (Figure 4C,D). In both cell lines, the treatment with osthole exerted the greatest effect, as it reduced the expression of this protein by 20% in MOGGCCM and 35% in T98G. Moreover, the application of sorafenib increased the level of this protein by 15% in the GBM cells. Better effects were evident in the case of PI3K (Figure 4E,F). The treatment with osthole, sorafenib, and the combination of both compounds decreased the level of this protein. The simultaneous application of the drugs was the most effective, as it caused an over 70% decrease in PI3K expression in both cell lines. The worst effect was observed upon the application of sorafenib alone, which reduced the PI3K levels by 25% in MOGGCCM and 15% in T98G.

#### 2.3.2. Level of Beclin 1

Beclin 1 induces the formation of an autophagosome, thereby initiating the process of autophagy. In the MOGGCCM line (Figure 4G), overexpression of this protein was visible after the treatment with osthole alone (a 40% increase) and in combination with sorafenib (over a 10% increase). In turn, the use of sorafenib alone was associated with a slight decrease in the expression. Completely different results were obtained in the T98G cell line (Figure 4H). Sorafenib increased the level of Beclin 1 by ca. 15%. The coumarin (alone and in combination with sorafenib) inhibited the expression of this protein.

### 2.4. Apoptosis, Autophagy, and Necrosis Induction Upon Inhibition of PI3, Beclin 1, and Bcl-2 Expression

#### 2.4.1. Blocking PI3K Expression

**Figure 5:**
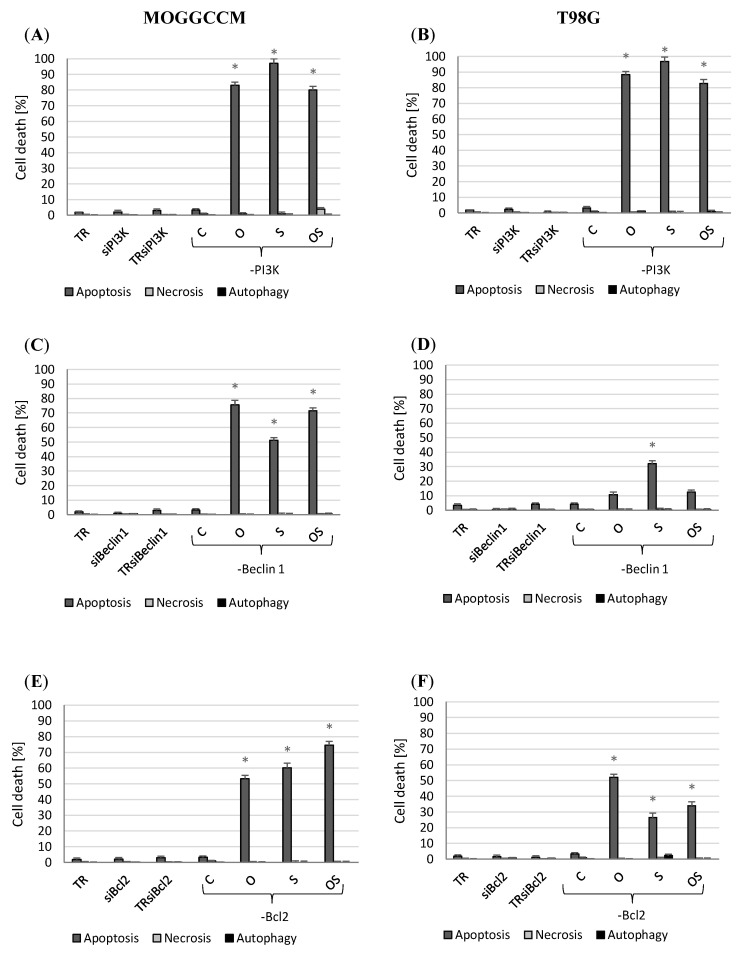
Level of apoptosis, autophagy, and necrosis in the MOGGCCM (**A**,**C**,**E**) and T98G (**B**,**D**,**F**) cells with inhibited PI3K (**A**,**B**), Beclin 1 (**C**,**D**), and Bcl-2 (B-cell lymphoma 2) (**E**,**F**) expression by specific siRNA (small interfering RNA) upon the osthole and sorafenib treatment. C—control, O—osthole, S—sorafenib; * *p* < 0.05.

The neoplastic transformation of gliomas is associated with excessive activation of the Ras/Raf/MEK/ERK and PI3K/Akt/mTOR pathways. Therefore, the studied cell lines were incubated with anti-PI3K siRNA and cells with blocked PI3K expression were additionally incubated with sorafenib. A significant increase in the sensitivity of the MOGGCCM and T98G cells to the induction of apoptosis was then observed (Figure 5A,B). The treatment with osthole and sorafenib, alone and in combination, induced apoptosis in at least 80% of cells. Sorafenib was the most effective agent leading to the death of almost all cancer cells (97%) in both cell lines. No autophagy or necrosis was observed in both cell lines.

#### 2.4.2. Inhibition of Beclin 1 and Bcl-2 Expression

The antiapoptotic protein Bcl-2 is responsible for the regulation of both apoptosis and autophagy. In a complex with Beclin 1, it inhibits autophagy and, after dissociation, disrupts apoptosis. Blocking the expression of Bcl-2 and Beclin 1 proteins in the AA cells inhibited autophagy and significantly increased apoptosis (Figure 5C,E). Osthole in combination with sorafenib exerted the most potent effect and induced apoptosis in over 70% of the cells. An increase in the apoptotic potential (up to 50%) was also observed in the GBM cells with blocked Bcl-2 expression upon the osthole treatment. In turn, the transfection had no effect on apoptosis induction in the treatment with sorafenib alone and in combination with the coumarin. However, it inhibited autophagy. The GBM cells with the blocked expression of Beclin 1 were less sensitive to induction of programmed death (Figure 5D,F). Apoptosis (30%), but not autophagy, was observed only after the sorafenib treatment.

### 2.5. Chou-Talalay Method—Effect of Combination Therapy

**Figure 6:**
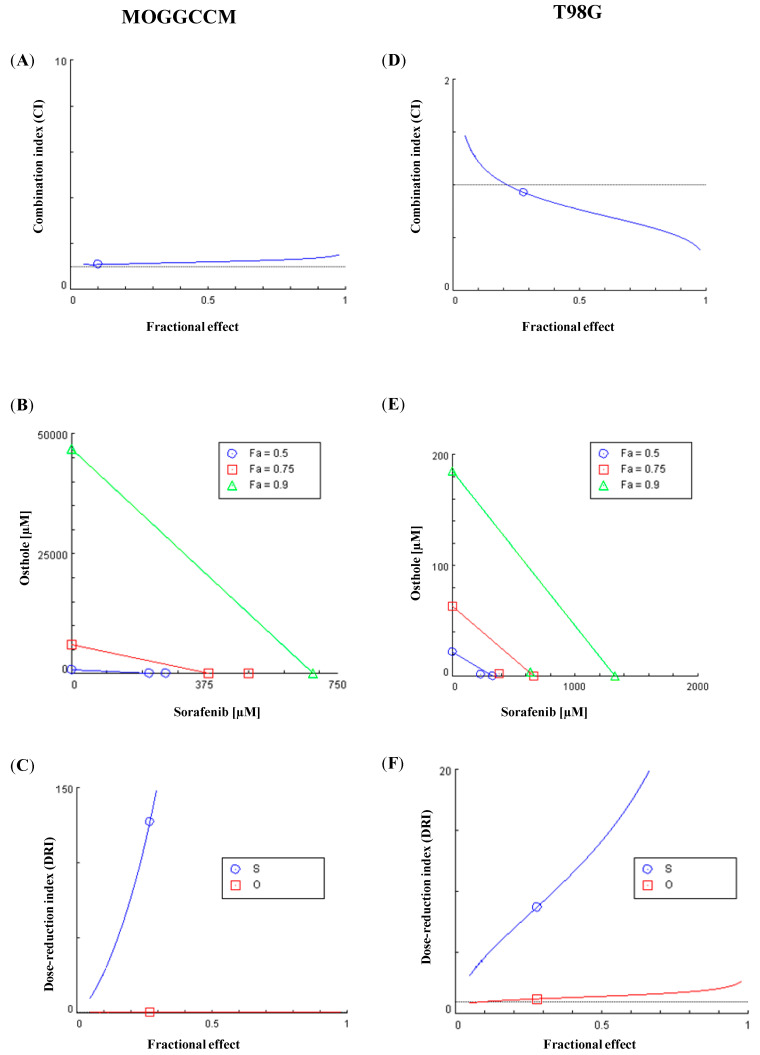
Osthole (O) and sorafenib (S) combination treatment in the MOGGCCM (**A**–**C**) and T98G (**D**–**F**) cell line. (**A**,**D**) Combination index (CI) plot: the combination index is plotted as a function of Fa (fractional effect, line of blue color). (**B**,**E**) Isobologram for the combination: classic isobologram at IC50, IC75, and IC90. (**C**,**F**) Fa-DRI (dose-reduction index) plot (Chou-Martin plot).

Drug interactions were determined using the isobologram, dose reduction, and combination index method derived from the median-effect principle proposed by Chou and Talalay [27]. As it turned out, the combination of osthole with sorafenib in the T98G line had a synergistic effect (Figure 6D–F). It was stronger when the higher dose was used, which is extremely important in anticancer therapy (for IC97, CI = 0.4). Moreover, the combination treatment significantly reduced the doses of both drugs (DRI > 1), which would have to be higher in a single application to yield the same effect. We observed different results in the MOGGCCM line, where the doses used had an additive effect (CI ≈ 1), while the higher drug concentrations were already antagonistic (Figure 6A–C). The effectiveness of the combination therapy was estimated on the basis of the ability to induce apoptosis. We also observed autophagy in this cell line, which was inhibited by the simultaneous treatment with osthole. For this reason, the use of combinations of these compounds is also appropriate in AA cells.

## 3. Discussion

Gliomas, i.e., tumors of the central nervous system, account for approx. 70% of all brain tumors. Due to their infiltrative nature, they are practically impossible to remove surgically [1,25]. Many resistance mechanisms are activated at the molecular level. An example is the Ras/Raf/MEK/ERK pathway. It has been shown that inhibition of this pathway reduces the survival, proliferation, migration, and metabolism of cancer cells [28,29,30]. It also reduces cancer resistance to the chemotherapy. Our previous studies have shown that sorafenib, i.e., a Raf kinase inhibitor, increases the apoptotic activity of Temozolomide and quercetin [31,32]. It has also been proved that simple coumarins: 4-hydroxycoumarin, umbelliferone, esculin, and osthole can play an important role in cancer therapy [33]. Therefore, in our studies, we used a combination of sorafenib as the Raf kinase inhibitor and the coumarins.

Our experiments showed that the anticancer properties of coumarin derivatives are closely related to their chemical structure. Osthole had the strongest apoptotic activity, alone and in combination with sorafenib. This compound has a methoxy and isopentenyl moiety attached to the C7 and C8 positions, respectively (Figure 1D). The other coumarins (4-hydroxycoumarin, umbelliferone, and esculin) are monohydroxy derivatives (Figure 1A–C). Additionally, esculin has a glycosidic substituent. It has been shown that the cytotoxic effect of coumarins is stronger with the increase in the hydrophobicity of the substituent, which is ensured by the isoprenyl group in osthole [34]. It has also been reported that the length of the substituted aliphatic chain has a great influence on the antitumor activity of the compound [35,36]. The enhanced lipophilicity of the alkyl group contributes to improvement of the ability of compounds to penetrate the cell membrane [37]. In the present experiment, there were no significant differences in the antitumor activity of umbelliferone and 4-hydroxycoumarin in both cell lines. Research conducted by Budzisz et al. showed similar effects. It was found that both hydroxycoumarins inhibited cell proliferation in a gastric carcinoma cell line with similar effectiveness [34]. Thus, the site of attachment of the hydroxy moiety (at the C4 or C7 position) does not significantly affect the proapoptotic properties of the coumarins. Moreover, the presence of an additional glycosidic moiety (esculin) did not change the properties of the compounds in the T98G line and increased the proapoptotic activity in the AA cells. Our experiments confirm observations described by other authors who reported that the anticancer effect of coumarins depends on both the chemical structure and the cell line used. The human carcinoma KB cell line was more sensitive to esculin treatment, while umbelliferone was more effective in HL60 cells [37]. In addition, the combined treatment with sorafenib decreased the sensitivity of the AA cells to the hydroxycoumarins (4-hydroxycoumarin and umbelliferone) treatment and increased the exposure to esculin.

Osthole, alone and in combination with sorafenib, induced apoptosis in approx. 30% of the glioblastoma cells. Our previous research showed that Temozolomide (TMZ)—a drug currently used to treat gliomas, induced apoptosis in 12% of the GBM cells and 5% of the AA cells [38,39]. Interestingly, another chemotherapeutic agent, also used in the treatment of gliomas, Bevacizumab (BEV), similarly to TMZ, reduces the GBM viability by approx. 15% [40]. Thus, our results suggest that the efficacy of osthole with sorafenib may be much higher than that of TMZ and BEV.

In our experiments, at the molecular level, the induction of apoptosis by osthole and sorafenib was accompanied by an increase in caspase 3 expression. On the other hand, the coumarin induced programmed death type I and II in the MOGGCCM cells. Moreover, the combination of both compounds reduced the number of apoptotic cells and completely inhibited autophagy. In this case, the elimination of the process of autophagy is desirable, as it can inhibit cell death in conditions of nutrient deficiency. Cells in the center of tumors are metabolically stressed in this manner and therefore, they use autophagy as a survival mechanism [41]. Blocking this process significantly increases the effectiveness of anticancer therapies used [42]. We also observed that the treatment with osthole alone was associated with overexpression of Beclin 1, which enhanced apoptosis in addition to autophagy. This was accompanied by an increased level of caspase 3. These results suggesting that Beclin 1 has both proautophagous and proapoptotic functions are consistent with studies conducted by Fururya et al. [43] and Huang et al. [44]. They showed that an increase in the expression of Beclin 1 in human gastric (MKN28) and glioma (U87) cells led to apoptosis by increasing the activity of caspase 3, 7, and 8. Interestingly, the treatment with osthole (alone and in combination with sorafenib) in the T98G cells with blocked Beclin 1 expression inhibited not only autophagy but also apoptosis. It has been shown that Beclin 1 can affect cell survival by interacting with Bcl-2 or Bcl-xL proteins. In turn, Bcl-2 may act as an antiautophagy protein by forming a complex with Beclin 1 [45]. Thus, after silencing the expression of the autophagy marker, the Bcl-2 protein inhibited apoptosis. Different effects were obtained in the AA cells, where instead of apoptosis reduction, we noted a significant increase in the percentage of apoptotic cells. Similar results were noticed after silencing the expression of the Bcl-2 protein. The percentage of apoptotic cells increased significantly after the combined treatment with sorafenib of the MOGGCCM cells. We did not observe autophagy. As with the Beclin 1 blocking, the T98G line was less sensitive to the treatment following the inhibition of Bcl-2 expression. The induction of apoptosis was also observed at that time, but the best effects were achieved only by the application of osthole. 

Glioblastoma cells often have mutations in the PTEN and PI3K genes, resulting in continued Akt/PKB kinase activity. This enzyme performs antiapoptotic functions, reducing the susceptibility of cells to inducers of this process and thus enabling tumor growth [25]. We noticed that the use of the combination therapy decreased PI3K protein expression in both cell lines, which correlated with the induction of programmed death. Moreover, blocking the expression of this protein significantly increased the effectiveness of the drugs used. Then, the treatment with sorafenib eliminated almost 100% of cancer cells. The coumarin (alone and in combination with sorafenib) exerted slightly worse effects, inducing over 80% cell death. Interestingly, blocking only PI3K kinase did not decrease the survival rate of the cancer cells. As it turned out, the inhibitors of this protein were also not cytotoxic in the single application; however, when combined with mTOR inhibitors, they significantly increased their effectiveness in eliminating gliomas [46]. A possible explanation is that PI3K inhibition induces other pathways that promote cancer cell survival [47]. Therefore, blocking PI3K expression in combination with osthole or sorafenib gave such good results. Our results suggest that the Ras/Raf/MEK/ERK and PI3K/Akt/mTOR pathways play a key role in the pathogenesis of grade III and IV gliomas.

We observed that the coumarin treatment led to a decrease in Raf kinase expression in both cell lines. At that time, the morphological analysis showed apoptosis, which is consistent with other literature reports. Raf kinase has antiapoptotic functions, hence a decrease in its activity promotes induction of apoptosis [48]. In turn, sorafenib led to the overexpression of this protein in the MOGGCCM line. The autophagy observed was a protective and adaptive response to the inhibition of the Raf/Raf/MEK/ERK pathway. This mechanism was described in many types of cancer, including brain cancer (B76, AM38, and BT40) [49], pancreas (MiaPaCa2 and BxPC3) [50], and melanoma (e.g., A375P, SKMEL5, 1205Lu, MEL624) [51]. It has also been shown that supporting targeted therapy through the use of autophagy inhibitors significantly increased the effectiveness of treatment [49,50]. Therefore, it can be assumed that, in addition to its proapoptotic properties, osthole has antiautophagy activity.

Gliomas have high migratory and invasive potential. Therefore, when planning new therapeutic strategies, drugs that inhibit the translocation of cancer cells should be considered. Our results indicated that the combination of osthole with sorafenib can be used for this purpose.

## 4. Materials and Methods 

### 4.1. Cells and Culture Conditions

Human glioblastoma multiforme cells (T98G, European Collection of Cell Cultures) and human anaplastic astrocytoma cells (MOGGCCM, European Collection of Cell Cultures) were grown in a 1:3 mixture of DMEM (Dulbecco’s modified Eagle medium) and nutrient mixture F-12 Ham (Ham’s F-12, Sigma). Both cell lines were cultured in medium supplemented with 10% FBS (fetal bovine serum) (Sigma), 100 U/mL penicillin (Sigma), and 100 mg/mL streptomycin (Sigma) at 37 °C in a humidified atmosphere consisting of 5% CO_2_ and 95% air. 

A primary culture of human skin fibroblasts (HSF), which was carried out in the same conditions as the tumor lines, was used in the study as well.

### 4.2. Coumarin Isolation

Osthole was obtained for the experiments after isolation from a petroleum extract of *Mutellina purpurea* L. fruits, with a method described previously [22] in the Independent Laboratory of Natural Products Chemistry, Medical University, Lublin, Poland. Two-phase solvent systems made of n-heptane, ethyl acetate, methanol, and water (HEMWat) with a volume ratio 3:2:3:2 were chosen as the most proper system for purification of target compounds (K = 1.8). After injection of 600 mg of a crude oily extract, 2 mg of the target compound were obtained. The identification of the isolated compound was carried out by comparison of the retention time and UV-DAD spectra with those obtained by standards in the same conditions. The purity of osthole was 99% (established with the HPLC-DAD method).

Umbelliferone was purified from fruits of *Heracleum leskowii* L. (Apiaceae) collected in the Medicinal Plant Garden, Department of Pharmacognosy with Medicinal Plant Unit, Medical University, Lublin, Poland in summer 2009, in accordance with a method published previously [15]. Briefly, the fruits were air-dried at room temperature and powdered, and a batch (100 g) was extracted with 100 mL of methanol under reflux for 30 min. After filtration, the procedure was repeated twice. The filtrates were combined and concentrated with a rotary evaporator to remove the solvent. The dried crude extract (13 g) was stored in a refrigerator until further separation. The Spectrum High-Performance Countercurrent Chromatograph (HPCCC) apparatus delivered by Dynamic Extractions (Slough, UK) was employed in the present study. The integrated analytical column (22 mL) was first entirely filled with the upper stationary phase. Then the apparatus was rotated at 200× *g* and the lower mobile phase was pumped into the column at a flow rate of 1.0 mL/min. After hydrodynamic equilibrium was reached, each time 30 mg of the extract dissolved in 1 mL of the two-phase solvent system was loaded onto the column through a 1 mL injection valve. When optimal conditions were determined, the procedure was transferred to a semipreparative integrated column (137 mL volume). The mobile phase was pumped at a flow rate of 6.0 mL/min and 180 mg of the extract was dissolved in 6 mL of the two-phase solvent system and loaded onto the column through a 6 mL injection valve. The solid-phase retention was 70%. The effluent from the column was continuously monitored with a UV detector at 320 nm (Ecom, Prague, Czech Republic). A mixture of n-heptane, ethyl acetate, methanol and water at a ratio of 1:2:1:2 was chosen for further experiments. Umbelliferone was isolated after 20 min. After injection of 180 mg of the crude extract, 1.8 mg of umbelliferone with 99% purity (according to HPLC analysis) was purified. All solvents for HPCCC and HPLC analysis were delivered by Avantor Performance Materials Poland S.A. (Gliwice, Poland—formerly POCh).

Esculin and 4-hydroxycoumarin were delivered by Sigma Aldrich. 

### 4.3. Drug Treatment

Sorafenib (Nexavar, BAY 43-9006) (1 µM), osthole (150 µM), and hydroxycoumarins: 4-hydroxycoumarin (Sigma), umbelliferone, and esculin (Sigma) at the final concentrations of 200 µM were used in the experiments. The drugs were dissolved in DMSO (Sigma) to the final concentration not exceeding 0.01%. The doses were chosen based on previous studies [32,33]. The cancer cells were treated with the coumarins or with sorafenib separately or in combination for 24 h. As controls, T98G and MOGGCCM cells were incubated only with 0.01% of DMSO.

### 4.4. Fluorescence Microscopy (Apoptosis, Necrosis, Autophagy Identification) 

For identification of apoptosis and necrosis, a solution of propidium iodide (Sigma, St. Louis, MO, USA) and Hoechst 33342 (Sigma, St. Louis, MO, USA) in distilled water in a ratio of 3:2:5 were used. The cells were stained upon incubation with the appropriate combinations of the drug. After addition of 2.5 µL of the mixture to 1 mL of the medium, the tumor cells were incubated at 37 °C for 5 min. The morphological analysis was performed under a fluorescence microscope (Nikon E—800, Tokyo, Japan). It showed bright blue fluorescence characteristic of apoptotic cells. Necrotic cells emitted red-pink fluorescence. Five percent acridine orange was used to identify autophagous cells incubated in the dark for 15 min. The dye induced the red glow of the autophagous bodies. At least 1000 cells in randomly selected microscopic fields were counted under the microscope. Each experiment was conducted in triplicate. 

### 4.5. Cell Migration Test

Tumor cell migration was assessed by means of the wound assay model [31]. The cell lines were grown at 2.5 × 105 in standard conditions (37 °C, 95% humidity, 5% CO_2_) in 4 cm-diameter culture dishes (NuncTM, ThermoFisher, Rochester, NY, USA). The next day, the cell monolayer was scratched with the pipette tip (P300), the medium and dislodged cells were aspirated, and the plates were rinsed twice with PBS. Next, fresh culture medium was applied and the number of cells that had migrated into the wound area after 24 h was estimated in the control and drug-treated cultures. The plates were stained with the May–Grünwald–Giemsa method. The observation was performed with the use of a BX51 microscope (Olympus, UK), and the distances between the scratch fronts were estimated using the CellSans program. The results were presented as the migratory potential expressed as the percent of cells within the wound. 

### 4.6. Western Blotting Analysis

The expression of cellular proteins was evaluated by Western blotting. After treatment for 24 h, cells grown in Falcon flasks (5 mL) were lysed in buffer (125 mM Tris-HCl pH 6.8; 4% SDS; 10% glycerol; 100 mM dithiothreitol). The cells prepared in this way were boiled for 10 min and then centrifuged at 12,000× *g* centrifugal force for 10 min; next, the supernatants were collected. The protein concentration in the cell-free extracts was determined with the Bradford method [52]. Equal amounts of protein (80 μg) from each sample were separated on SDS-PAGE (SDS poliacrylamide gel electrophoresis) [53] and transferred onto an Immmobilon P membrane (Sigma). After blocking with 3% low fat milk for 1 h, the membranes were incubated overnight with primary antibodies: rabbit anti-caspase 3 (Sigma, 1:1000), anti-Beclin 1 (Santa Cruz Biotechnology, 1:500), anti-Raf (Santa Cruz Biotechnology, 1:500), and anti-PI3K (Santa Cruz Biotechnology,1:500). After three washes with PBS enriched with 0.05% Triton X-100 (Sigma), the membranes were incubated with secondary antibodies conjugated with alkaline phosphatase (AP) for 2 h. Alkaline phosphatase substrates: 5-bromo-4-chloro-3-indolylphosphate (BCIP) and nitro-blue tetrazolium (NBT) (Sigma) in *N*,*N*-dimethylformamide (DMF, Sigma) were used for visualization of proteins (Bcl-2, Beclin 1, and caspase 3). The results were analyzed qualitatively on the basis of the band thickness, width, and color depth. The quantitative analysis of protein bands was performed using the Bio-Profil Bio-1D Windows Application V.99.03 program. The data were normalized relative to β-actin (Sigma, working dilution 1:2000). Three independent experiments were performed.

### 4.7. Cell Transfection

The cells at a density of 2 × 10^5^ were incubated for 24 h at 37 °C in a CO_2_ incubator to reach 60–80% of confluence. The cells were washed with a DMEM:Ham’s F-12 (3;1) mixture without serum and antibiotics and aspirated. Next, a blocking mixture containing 2 µL of specific anti-PI3K, anti-Bcl-2, or anti-BCN1 siRNA (Santa Cruz Biotech Dallas, TX, USA), 2 µL of Transfection Reagent (Santa Cruz Biotech, Dallas, TX, USA), and 250 µL of Transfection Medium (Santa Cruz Biotech) was added. The cells were incubated for 5 h at 37 °C, 5% CO_2_, and 95% humidity. Next, the medium was supplemented with medium containing 20% of fetal bovine serum and 200 µg/mL of antibiotics. After 18 h, the medium was replaced with a fresh one (containing 10% FBS, 100 U/mL penicillin, and 100 mg/mL streptomycin) and the transfected cells were used for further studies (incubation with osthole and sorafenib alone and in combination as well as determination of cell death).

### 4.8. Statistical Analysis

A one-way anova test followed by Dunnett’s multiple comparison analysis was used for statistical evaluation. *p* < 0.05 of data presented as mean ± standard deviation (SD) was taken as the criterion of significance.

### 4.9. Chou-Talalay Method

The combination index (CI) and the dose reduction index (DRI) were calculated with the method developed by Chou and Talalay [23] using the Compusyn software and the original data of programmed cell death induction in the MOGGCCM and T98G cells upon the sorafenib or osthole treatment [32,33]. CI < 1, CI = 1, and CI > 1 indicate a synergistic, additive, and antagonistic effect, respectively. The DRI represents the fold reduction of compounds as a result of the synergistic combination compared to the concentration of the drug alone required to reach the same effect.

## 5. Conclusions

Due to their widespread availability and the broad spectrum of biological activity, coumarins have enormous pharmacological potential. Their anticancer properties, which depend on the chemical structure of the compounds, deserve special attention. Our research has shown that the presence of an isoprenyl moiety (osthole) significantly increases this activity, compared to the other coumarins. It also sensitizes glioma cells with a decreased level of PI3K to apoptosis induction in combination therapy with sorafenib. Therefore, the present results may therefore constitute a basis for further research on the development of new anticancer therapies.
